# Recent updates on CAR T clinical trials for multiple myeloma

**DOI:** 10.1186/s12943-019-1092-1

**Published:** 2019-11-05

**Authors:** Quande Lin, Juanjuan Zhao, Yongping Song, Delong Liu

**Affiliations:** The Affiliated Cancer Hospital of Zhengzhou University and Henan Cancer Hospital, 127 Dongming Road, Zhengzhou, 450008 China

**Keywords:** B cell maturation antigen, BCMA, Chimeric antigen receptor, CAR T, Multiple myeloma

## Abstract

Proteasome inhibitors, immunomodulatory agents and monoclonal antibodies have dramatically changed the natural history of multiple myeloma (MM). However, most patients eventually suffer a relapse and succumb to the disease. Chimeric antigen receptor (CAR) engineered T cells targeting B cell maturation antigen (BCMA), CD138, CS1 glycoprotein antigen (SLAMF7) and light chains are in active development for therapy of refractory /relapsed (RR) MM. CD19- targeted CAR T cells in conjunction with autologous stem cell transplantation also showed activity in RRMM. Dual- target CAR T cells are in clinical trials for RRMM. This review summarized the recent updates of ongoing CAR T clinical trials for multiple myeloma.

## Background

Proteasome inhibitors, immunomodulatory agents (IMiDs) and monoclonal antibodies have dramatically changed the natural history of multiple myeloma (MM) [1–7]. However, most patients eventually suffer a relapse and succumb to the disease after multiple lines of therapy [8–11]. Chimeric antigen receptors (CARs) are engineered receptors that can bind to a desired antigen and redirect the effector cells to a defined target [12–17]. Two CD19-engineered CAR T cell products, axicabtagen ciloleucel (yescarta, Kite) and tisagenlecleucel (kymriah, Novartis), have been approved for therapy of advanced B cell malignancies. The major clinical toxicities of CAR T cell therapy are cytokine release syndrome (CRS) and CAR T- related encephalopathy syndrome (CRES) [18–20], which require prompt diagnosis and intervention to prevent fatal complications [21, 22]. The CD19 targeted CAR-T cell therapy has inspired tremendous interests in searching for new targets for MM immunotherapy [23–30]. There are various ongoing clinical trials using CAR T cell technology to target myeloma antigens such as B cell maturation antigen (BCMA), CD138, CS1 glycoprotein antigen (SLAMF7) and immunoglobulin light chains [31–35] (Table 1). This review summarized ongoing CAR T clinical trials for multiple myeloma.

**Table 1:** BCMA-targeted CAR T clinical trials in multiple myeloma

Registration number (reference)	Phase	Dosage	No. of patients	Responses
NCT02215967 (49)	1	0.3–9.0 × 10^6^ CAR+T cells/kg	12	sCR: 1; VGPR:2; PR:1; SD:8
NCT02658929 (51)	1	50–800 × 10^6^ CAR+ T cells	33	sCR:12; CR:3; VGPR:9; PR:4; SD:4; PD:1
NCT03274219 (52)	1	150 × 10^6^ CAR+ T cells	8	sCR: 1; VGPR: 3; PR: 2 MRD negative: 3
NCT03090659 (55)	1/2	0.07–2.1 × 10^6^ CAR+ T cells/kg	57	CR: 39; VGPR: 3; PR: 8; MRD negative: 36; ORR: 88%
ChiCTR-ONH-17012285 (53)	1	0.21–1.52 × 10^6^ CAR+ T cells/kg	17	sCR: 13; VGPR: 2; ORR: 88.2% NR: 1
NCT03430011 (58)	1/2	50–150 × 10^6^ CAR+ T cells	19	sCR: 2; CR: 1; VGPR: 2; PR: 2; MR: 1
NCT03915184 (59)	NA	0.5–1.8 × 10^8^ CAR+ T cells	16	CR: 2; PR: 4; VGPR: 6 ORR: 100%
NCT03070327 (61)	1	72–818 × 10^6^ CAR+ T cells	11	VGPR: 2; ORR: 64%
ChiCTR1800018137 (64)	1	1.0–6.0 × 10^6^ CAR+ T cells/kg	9	CR:4; VGPR:1; PR: 4; ORR: 100%
NCT02546167 (65)	1	1–50 × 10^7^ CAR+T cells	25	sCR: 1; CR: 1; VGPR: 5; PR: 5
NCT03288493 (67)	1	48–430 × 10^6^ CAR+ T cells	12	sCR: 1; nCR: 1; VGPR: 1; PR: 2
NCT03338972 (69)	1	5–15 × 10^7^ CAR+ T cells	7	ORR:100%

*Abbreviations: BCMA* B cell maturation antigen, *CAR* Chimeric antigen receptor, *VGPR* Very good partial response, *SD* Stable disease, *CR* Complete response, *PR* Partial response, *sCR* Stringent complete response, *PD* Progressive disease, *ORR* Overall response rate, *MRD* Minimal residual disease, *nCR* near Complete response, *BMPC* Bone marrow plasma cells, *IHC* Immunohistochemistry, *FC* Flow cytometry, *MR* Minimal response, *NR* non-response, *RRMM* relapsed/refractory Multiple Myeloma, *NA* not available, *E* evaluable

## BCMA (B cell maturation antigen)

BCMA was discovered initially by several groups [36–39]. BCMA gene was found to be fused to the interleukin-2 gene in the t(4;16) (q26;p13) translocation in a malignant T-cell lymphoma. BCMA gene is localized on chromosome band 16p13.13. The BCMA gene encodes a peptide with 184 amino acid residues and an estimated molecular weight of 20kd [37].

BCMA is also known as CD269 and TNF receptor superfamily 17 (TNFRSF17) [40]. BCMA ligands include B cell-activating factor (BAFF, also termed TNFSF13B) and a proliferation- inducing ligand (APRIL, also termed TNFSF13) [41]. BCMA is expressed almost exclusively in B lineage cells including plasmablasts and in particular at the stage from mature B to plasma cell (PC) terminal differentiation. In addition to normal B cells, BCMA is also expressed on MM cells and malignant B cells [31, 42]. BCMA is known to be absent on naïve and most memory B cells. In BCMA knock-out mice it was shown that the mice had normal B cell development and an intact humoral immune system [43]. BCMA expression is upregulated during PC differentiation. Hence, even though BCMA may not be critical for B-cell development, it plays a major role in B-cell maturation and differentiation into plasma cells. BCMA appears to enhance the survival of normal PCs and plasmablasts as well as long-lived PCs in the BM.

BCMA has a soluble form found in the peripheral blood of MM patients [44]. Injection of the soluble BCMA disrupted immune responses, affected splenic architecture and prevented the accumulation of peripheral B cells [45–47]. The soluble BCMA therefore may interfere theoretically with the myeloma-targeting capacities of BCMA-specific immunotherapeutics [48].

## BCMA-targeted CAR T cell trials

### Early BCMA-targeted CAR T trial

**Fig. 1:**
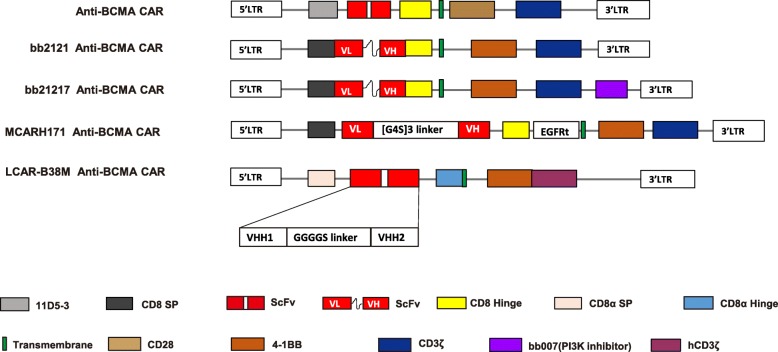
The schematic diagram of representative structures of BCMA-targeted chimeric antigen receptors (CAR). The BCMA CARs contain a single-chain of BCMA antibody variable fragment (ScFv), a transmembrane domain, a hinge region, a co-stimulation domain (4-1BB, CD28 or OX40), and a CD3z domain. Additional sequences (such as PI3K inhibitor) are added to enhance identification of CAR+ T cells. LCAR-B38M CAR contains two epitopes of BCMA ScFv, VHH1 and VHH2. PI3K: phosphoinositol 3 kinase

In a study of cell lines and human tissues, BCMA was found to be expressed in plasma cells and myeloma cells, but not in normal tissues and neither in hematopoietic stem cells. The first BCMA CAR contained a CD28 co-stimulation domain [31] (Fig. 1). The first-in-human phase I clinical trial of CAR T cells targeting BCMA was conducted in patients with RRMM (NCT02215967) [49]. Twelve patients were reported in the dose escalation trial. Four dose levels were reported. The four levels were 0.3, 1.0, 3.0, 9.0 × 10^6^/kg. Among the 12 patients, 3 patients entered partial remission (PR), 8 patients had stable disease (SD), and 1 patient achieved stringent complete remission (sCR). Among the 6 patients treated on the 2 lowest dose levels, limited anti-myeloma activity and mild toxicity occurred. On the third dose level, 1 patient obtained a very good PR (VGPR). Two patients were treated on the fourth dose level of 9 × 10^6^ CAR T cells/kg. After treatment, bone marrow plasma cells of the two patients became undetectable by flow cytometry. The first patient entered a sCR that lasted for 17 weeks before relapse, and the serum monoclonal protein of the second patient had decreased by > 95% 28 weeks after infusion of CAR-BCMA T cells. This patient remained in an ongoing VGPR. Both patients treated on the fourth dose level had CRS. The patients who received higher doses of CAR T cells had better responses but also a higher risk for adverse events (AEs), including CRS. This study also noted that soluble BCMA did not interfere with the efficacy of the BCMA-targeted CAR T cells. In addition, decrease of the soluble BCMA in the serum may serve as a biomarker for the efficacy of the anti-BCMA CAR T cells. This study was significant for the proof of concept of BCMA as a unique target for plasma cell malignancies.

In a follow-up report, 16 patients with RRMM were treated at the highest dose level of 9 × 10^6^ CAR T cells/kg [42]. Among these 16 patients, 81% responded, with 63% VGPR or CR. Median event-free survival (EFS) was 31 weeks. In addition to eradication of bone marrow myeloma cells, soft-tissue plasmacytomas were also eliminated. Negative minimal residual disease (MRD) was achieved in the bone marrow in 11 responders.

### bb2121 anti-BCMA CAR T cell trials

Two types of BCMA-targeted CAR T cells from Blue Bio were reported, bb2121 and bb21217. Bb2121 contains a 2nd generation CAR with 4-1BB co-stimulation domain. Based on the bb2121 CAR structure, bb21217 contains an extra domain of bb007 which encodes a PI3K inhibitor [50](Fig. 1). This makes it possible to select out the BCMA CAR engineered T cells.

bb2121 was studied in a phase 1, single infusion dose escalation trial in patients with RRMM (CRB-401, NCT-2658929) [51]. Four dose levels were infused at 50 × 10^6^, 150 × 10^6^, 450 × 10^6^, or 800 × 10^6^ CAR-positive (CAR+) T cells. Two doses at 150 × 10^6^ to 450 × 10^6^ CAR+ T cells were given in the expansion phase. Patients had failed at least 3 lines of therapy. Safety was the primary endpoint. A total of 33 patients were treated with bb2121. The most common severe adverse events (SAE) were cytopenia. 25 out of 33 patients (76%) had CRS grade 1–2, and 2 at grade 3. CRES was seen in 42% of the patients, grade 1–2 in all but one who had grade 4. The ORR was 85%, with 45% CR (*n* = 15). Six of the CR patients relapsed. The PFS was 11.8 months (6.2–17.7). Sixteen patients were MRD negative (≤10^− 4^ nucleated cells). The bb2121 CAR T cells remained detectable up to 1 year after infusion. It was noted that CAR T cell expansion correlated with clinical response.

CRB-402 (NCT03274219) is a first-in-human, multi-center phase I dose escalation trial of bb21217 CAR T cells in RRMM patients [52]. The study was designed to assess the safety, efficacy and duration of effect of bb21217. Patients with strong BCMA expression on MM cells (≥ 50% BCMA expression by immunohistochemistry) were enrolled. Four dose levels are planned: 150, 450, 800, and 1200 × 10^6^ CAR+ T cells. Safety was the primary endpoint. In the initial report, 8 patients have received a dose of 150 × 10^6^ CAR T cells, and 7 patients were evaluable for initial (1-month) clinical response. Five of the 8 patients had CRS. As of data cut-off, 6 of 7 patients had demonstrated clinical response per IMWG criteria: 1 sCR, 3 VGPR, 2 PR. MRD negative results were confirmed by next-generation sequencing (NGS) in 3 of 3 evaluable responders. The CAR T cells were detectable at 6 months post-infusion.

### LCAR-B38M: biepitopic targeting of BCMA

LCAR-B38M CAR T cells contain a CAR construct with scFv targeting two BCMA epitopes, VHH1 and VHH2 [53–55]. The BCMA-specific LCAR-B38M CAR T cells were initially tested in RRMM patients, some with extramedullary involvement [56]. In addition to objective responses, the subcutaneous mass in 2 patients was significantly shrunken after treatment. The most common adverse effects were CRS. No dose-limiting toxicities (DLTs) or treatment-related deaths occurred in these initial reports.

Results of the LCAR-B38M CAR T trial for RRMM (LEGEND-2, NCT03090659) was updated in 2018 in 57 patients [54, 55]. In this cohort, single-agent cyclophosphamide was used for lymphodepletion. The total dose of LCAR-B38M CAR T cells was divided into 3 infusions: 20, 30, and 50% of the total dose. The median CAR T cell dose was 0.5 × 10^6^ cells/kg [range, 0.07–2.1 × 10^6^]). In this report, 57 patients have been infused with LCAR-B38M CAR T cells. The overall response rate (ORR = PR or better) was 88%. CR rate was achieved in 39 patients (68%), VGPR was achieved in 3 patients, and PR was achieved in 8 patients. MRD was negative in 36 patients. The median time to initial response was 1 month. No clear relationship between LCAR-B38M CAR T cell dose and response was observed. BCMA expression did not correlate with clinical response. CRS was mostly grade 1 (47%) and 2 (35%); 4 patients (7%) had grade 3 CRS. No clear relationship was demonstrated between the CAR T cell dose and CRS.

In a separate report, the LCAR-B38M CAR T cells were given to 17 RRMM patients after lymphodepletion with cyclophosphamide and fludarabine [53]. In this report, two infusion schedules were compared, three infusions (*n* = 8) versus one infusion (*n* = 9) of the total CAR T dose. No differences in response were observed among the two delivery subgroups. Toxicities between the two subgroups were quite similar. Therefore, the two groups of patients (*n* = 17) were analyzed together. The median follow-up was 417 days. The ORR was 88.2%, including 13 sCR, 2 VGPR, 1 non-responder and 1 toxic death. Eight patients remained progression-free, 6 relapsed, and 1 progressed after VGPR. CAR T cell level correlated with disease status, with low level being associated with relapse /progression. It was also noted that anti-CAR antibody was responsible for high risk of relapse /progression. Consistent with previous observations, prior ASCT correlated with more durable response.

The outcome from the trial of 74 patients after LCAR-B38M CAR T therapy suggests that the biepitopic CAR T cells against BCMA can be an important type of immunotherapeutics for RRMM. Development of anti-CAR antibody requires attention in future trials. The second-generation human BCMA-targeted CAR may avoid this drawback.

### JCARH125

JCARH125 is a CAR T cell product containing a lentiviral BCMA- targeted CAR construct with an optimized spacer and a 4-1BB co-stimulatory domain [57]. This CAR T cell product is being evaluated in a multi-center phase I/II clinical trial, the EVOLVE (NCT03430011) trial in RRMM patients who have exhausted therapies [58]. A single dose of JCARH125 is scheduled on day 1 in each cohort. Dose escalation was done with the first 2 dose levels at 50 and 150 × 10^6^ CAR+ T cells. In this early report, 19 patients have been enrolled and leukapheresis completed. Among these, 13 patients received JCARH125 cells. Eight patients were evaluable for toxicities, 6 of which had grade I/II CRS. Three of the 8 patients had CRES. Objective response was seen in all 8 patients. However, the follow-up was very short, longer follow-up and more patients are needed. The study remains active.

### CT053

CT053 are genetically modified T cells with a human BCMA scFv and 4-1BB costimulatory motif. CT053 CAR T cells were studied in a multi-center investigator-initiated clinical trial in patients with RRMM (NCT03915184). BCMA expression was seen on MM cells in all patients. A single dose of CAR T cells is planed and a second dose is allowed as clinically indicated. In a recent report, 16 patients received CT053 cells [59]. CRS was seen in 3 patients, no CRES nor DLT was observed. With median follow-up time of 8 (4 to 36) weeks, ORR was 100% in the 13 evaluable patients. The CT053 CAR-T cells were detectable up to 4–6 months in 11/13 patients. CT053 BCMA-targeted CAR T cells appeared to have the potential for further development for RRMM.

### MCARH171

MCARH171 has a second generation, human derived BCMA targeted CAR which contains a 4-1BB domain and a truncated epidermal growth factor receptor safety system [60]. MCARH171 CAR T cells were studied in a phase I clinical trial and the final result of the phase I trial was updated at the 2018 ASH annual meeting [61]. The trial followed a standard 3 + 3 design. The 11 enrolled RRMM patients received one of the following doses per cohort: (1) 72 × 10^6^, (2) 137 × 10^6^, (3) 475 × 10^6^, (4) 818 × 10^6^ viable CAR+ T cells. Safety and efficacy as well as the persistence of CAR T cells were evaluated. The ORR was 64% and the median duration of response was 106 days (range: 17 to 235 days). Results indicated the peak amplification and persistence of MCARH171. Durable clinical responses were dose dependent. Patients treated on the first two cohorts (≤150 × 10^6^ CAR T cells) had a lower peak peripheral blood expansion (mean 14,098 copies/μL; *N* = 6), compared with patients in the cohort 3 and 4 (≥450 × 10^6^ CAR T cells; *N* = 5), where the average peak expansion was 90,208 copies/μL (*p* < 0.05). The response rate was 100% in 5 patients receiving higher doses (450 × 10^6^). The duration of response was also related to the cell dose, with 3 of 5 patients (60%) treated in the cohorts ≥450 × 10^6^ had a clinical response lasting > 6 months, compared with only 1 of 6 (16.7%) patients who received lower doses. Two patients continued to respond with VGPR during follow-up of 7.5 and more than 10 months respectively. As shown in this study, MCARH171 has acceptable safety and no DLT has been reported. The dose-response relationship with toxicity was not clearly observed. However, a dose-response relationship was observed with promising clinical efficacy at dose levels of ≥450 × 10^6^ CAR T cells.

### CT103A

Another BCMA – targeted CAR T cell product contained a lentiviral CAR with a murine anti-BCMA scFv and CD28z domain (BRD015) [62]. In the phase I trial, the target dose levels of anti-BCMA CAR T cells ranged between 5.4 ~ 25.0 × 10^6^ cells /kg. Among 28 evaluable patients, 26 achieved remission. Twenty-two patients had strong BCMA expression (> = 50% expression rate) on MM cells, whereas 6 cases had weaker BCMA expression. ORR was 87% for strong expressors, 100% for weak expressors. The OS for the strong expressors was not yet reached at the time of report, whereas the OS for the weak expressors was 206 days (*p* = 0.0468). It was observed that higher peak level of CAR T cells in the blood was associated with better responses. A patient with POEMS syndrome responded to the BCMA-targeted CAR T therapy [62, 63].

This BRD015 product has the murine BCMA epitope. Human blocking antibodies against murine antigen makes it ineffective for the CAR T cell re-infusion. To conquer this issue, a novel BCMA-targeted CAR-T, CT103A, was engineered, which contains a lentiviral vector with a fully human BCMA scFv and a 4-1BB co-stimulatory domain. In the latest update, a single-center, dose-escalating phase I trial of CT103A in patients with RRMM enrolled 9 patients, including 3 patients who have relapsed after the murine BCMA-targeted CART, BRD015, therapy (clinical trial registration: ChiCTR1800018137) [64]. The CT103A CAR T cells were administered following a standard 3 + 3 dose-escalation design, with three doses at 1, 3, and 6 × 10^6^ cells/kg. The conditioning chemotherapy regimen was cyclophosphamide and fludarabine. The median prior lines of therapy of the enrolled patients were 4 (range 3–5). The ORR was 100%. The response was observed within 14 days. In the first two dose levels, the CRS was mild. It is intriguing that among the three patients who relapsed after the murine BCMA CAR T therapy, two patients achieved CR and one patient achieved VGPR after CT103A therapy. Further studies are ongoing for this promising humanized BCMA- targeted CAR T therapy in RRMM.

### Cart-BCMA

Another autologous T cell product, CART-BCMA, containing a fully human, BCMA-specific CAR with CD3ζ and 4-1BB signaling domains was studied in a phase I clinical trial (NCT02546167) for RRMM patients (Table 1). This was a single-center standard 3 + 3 dose-escalation study with 3 cohorts, with doses ranging between 1.0 to 50 × 10^7^ total CART-BCMA cells. The cohort I group had no lymphodepletion therapy, whereas cohort 2 and 3 had cyclophosphamide for lymphodepletion. In the updated reports, 25 subjects were enrolled [65]. Among these, 8 had severe CRS and 3 had severe CRES respectively. One patient died with candidemia, severe CRS and encephalopathy. Responses were seen in all cohorts, including 5 PR, 5 VGPR, and 2 CR. There was correlation of responses and CART-BCMA expansion with CD4/CD8 T cell ratio and frequency of CD45RO^−^CD27^+^CD8^+^ T cells in the initial leukapheresis product. In this study, higher dose of CART-BCMA cells without lymphodepleting chemotherapy were tested in cohort 1. The CAR T cells were shown to be clinical active in heavily pretreated patients with MM in all cohort groups, with or without lymphodepletion chemotherapy. More subjects are being enrolled in expansion cohorts.

### P-BCMA-101

P-BCMA-101 encodes a CARTyrin that can target BCMA. CARTyrin is a non-immunoglobulin- based scaffold Centyrin molecule produced with a novel non-viral piggyBac transposon-based delivery system [66]. The Centryins are fully human with high binding affinities. These Centyrins are therefore less immunogenic. P-BCMA-101 is hence a novel CAR T product that can target BCMA. Since this is not based on a viral vector system, it is less costly. This approach also allowed manufacture of CAR T cells with predominantly more favorable stem cell memory T phenotype (T_SCM_). P-BCMA-101 is being studied in a phase 1, 3 + 3, single-administration dose escalation trial in patients with RRMM (NCT03288493) (Table 1). In the update at the 2018 ASH meeting, 12 high-risk heavily pre-treated patients with RRMM have been treated with P-BCMA-101 CAR-T cells in 3 cohorts [67]. Grade 2 CRS was observed in one patient. The treatment was well tolerated with no death and no CRES. DLT was not yet observed. These were consistent with the improved therapeutic index for these T_scm_ CAR T cells. The ORR was 83% in the evaluable patients.

### BCMA CAR T cells with defined formulation (CD4+:CD8+ =1:1)

It has been reported that CAR T cells with defined proportion of CD4 and CD8 cells may have advantages [68]. A first-in-human phase I trial of BCMA CAR T cells with defined formulation was done in RRMM patients (NCT03338972) [69]. In this study, CD8+ and CD4+ T cells were isolated, enriched and cryopreserved separately. These T cells were transfected with a fully human BCMA scFv -containing CAR via a lentiviral vector. The cells were expanded in the culture and the cell product for infusion was formulated to contain equal number of CD4+ and CD8+ BCMA CAR T cells. To facilitate tracking of the BCMA-targeting CAR T cells, a truncated non-functional human epidermal growth factor receptor (EGFRt) was inserted into the CAR cassette. The first dose level was 5 × 10^7^ EGFRt+ T cells (cohort A, *n* = 5). At the time of this report, 2 patients have been enrolled in the cohort B at the dose of 15 × 10^7^ EGFRt+ T cells. These seven patients have received a median of 8 prior regimens (range 6 to 11), including autologous stem cell transplant (SCT) (71%) and allogeneic SCT (43%). All seven patients had a response at 28 days. The EGFRt+ CAR T cells were still detectable 90 days after infusion. The median survival was 16 wks (range 2 to 26 wks) with all patients alive. One relapsed patient was found to become BCMA negative in the myeloma cells. At the time of the update, DLT and CRES have not been observed. Only grade 2 or lower CRS was reported. In summary, BCMA-targeted CAR T cells with a 1:1 ratio of CD4+:CD8+ were well tolerated and were shown to be effective at total cell doses as low as 5 × 10^7^. This approach deserves further investigation. Longer follow-up is needed.

## CD19- targeted CAR-T cell trials for MM

Tisagenlecleucel has been approved for advanced B cell acute lymphoid leukemia (ALL) and diffuse large cell lymphoma [24–28, 70–77]. CD19 expression is lost in plasma cells [78]. However, minor subsets of myeloma cells with unique propagating properties were found to express low levels of CD19. CR was reported in a case of RRMM patient after treatment with tisagenlecleucel cells (CTL019) following high dose melphalan (140 mg /m^2^) and autologous stem-cell transplantation (ASCT) [79]. The dramatic response was surprising and intriguing since there lacked CD19 expression in 99.95% of myeloma cells in this patient. The durable response continued even after disappearance of CTL019 cells in the blood (day 2 to day 47 post CTL019 infusion), suggesting that the sustained response did not require the persistent presence of the CAR T cells. This therapeutic approach of RRMM is being further investigated in a clinical trial (NCT02135406). A recent update detailed treatment of 10 patients with RRMM with CTL019 following high-dose melphalan and ASCT [32]. Eleven patients were enrolled at the time of the report, though T cells from one patient failed to proliferate to the required number. These 10 patients had previously undergone ASCT but progressed within 1 year. The treatment with ASCT + CTL019 was safe and feasible. There was no severe CRS, correlating with low concentration of B cells in the peripheral blood of these patients. The most toxicity observed was attributed to ASCT. In this study, 4 patients achieved objective response (sCR, *n* = 1, VGPR, n = 1, PR, *n* = 2), and other 6 objects remained progression free. Durable progression- free survival (PFS) after ASCT + CTL019 was reported in 2 of 10 subjects. Peak frequency of CTL019 cells in bone marrow and emergence of immune responses against the stem-cell antigen Sox2 correlated with the favorable clinical outcome. The two patients with the best responses had significant elevation of anti-Sox2 antibodies. Sox2 expression has been shown to correlate with the myeloma-propagating capability in myeloma cell lines [80–82]. It appears that CTL019 cells in this setting induced the immune responses against Sox2. This intriguing phenomenon may be related to epitope- spreading since CD19 and Sox2 are co-expressed in myeloma-propagating cells (MPC) which are targeted by CTL019 cells [32]. This possible MPC targeting mechanism was further investigated ex vivo. It was demonstrated that MPCs as colony forming cells were reliably targeted by the combination of CTL019 and anti-BCMA CAR T cells, since CTL019 and anti-BCMA CAR T cells each can target a subset of MPCs. These observations suggest that the surface immunophenotypes of MPCs are heterogeneous, some resembling CD19+ B cells, some resembling BCMA+ plasma cells.

## Cocktail strategy of BCMA- and CD19- targeted CAR T trials for MM

To combat antigen loss and resistance of CAR T therapies, combining CAR T cells with different target in a cocktail infusion has been reported [83–85]. One report evaluated the safety and efficacy of combined infusion of CD19- and BCMA- targeted CAR T Cells for RRMM (NCT 03196414) [86]. The CARs contained anti- BCMA or anti-CD19 single chain variable fragment (scFv), the cytoplasmic portion of the OX40 and CD28 costimulatory moiety, and the CD3z T-cell activation domain. These are third generation CARs. CAR T-19 cells were infused on day 0 at the dose of 1 × 10^7^/kg, and CAR T-BCMA cells were given as split-dose infusions (40% on day 1 and 60% on day 2). Two of the 8 patients received haplo-identical BCMA targeted CAR T cells. All 8 patients had CRS. Due to the several compounding factors (such as haplo-identical T cells) in this trial, it is difficult to evaluate the outcome of this small combined CAR T clinical trial in RRMM.

Using the same strategy of cocktail CAR T therapies, the same group reported infusion of BCMA- and CD19-targeted CAR T cells into RRMM patients on day 14 to day 20 following autologous stem cell transplantation (SZ-MM-CART02 study, NCT 03455972) [87]. The CAR T cells were thereby used as post-transplant consolidation therapy. The median follow-up was only 3 months (2–11 months) at the time of report with 9 patients enrolled in cohort 1. All patients were positive for BCMA and negative for CD19 expression on MM cells. The ORR was 100%, with 3 CR, 6 VGPR after CAR T therapy. MRD negativity in BM was 37.5% after transplantation which increased to 66.7% after CAR T therapy. All patients experienced mild grade 1–2 CRS. There was more than 1000-fold expansion observed at peak level. A 100- fold increase was reported in a similar cohort. This phenomenon is intriguing, since it has been postulated that CD19 CAR may enhance T cell expansion in vivo at the expenses of B cell hypoplasia. Presence of the CD19- targeted CAR T cells in the cocktail may be responsible for the significant expansion of the CAR T cells. As discussed above, CTL019 infusion after ASCT can induce CR. It is therefore not clear what proportion of the response in the cocktail CAR T therapy after ASCT in this study was attributable to the cocktail vs to the CD19-targeted CAR T. Theoretically, the strategy of cocktail infusion of CAR T cells may inherently lead to higher toxicities due to simultaneous targeting of two or more antigens /epitopes and therefore enhanced T cell activation and tumor lysis. From these early studies of cocktail CAR T cell therapy, it is still difficult to make conclusions. More studies with larger sample sizes are needed. CRS as the main AE is better recognized and managed [18–20]. Recent recognition of roles of macrophage and monocyte in CRS and CRES may lead to earlier and better therapy as well as possible prophylaxis.

## SLAMF7-targeted CAR T trials

SLAMF7 (also known as CS1) is an antigen abundantly expressed on the surface of normal and neoplastic plasma cells and NK cells as well as a small subset of lymphocytes [88–94]. Elotuzumab (huLuc63) is a monoclonal antibody against SLAMF7. In clinical trials, elotuzumab in combination with IMiDs and proteasome inhibitors has been shown to be highly effective in treating RRMM [95]. Elotuzumab has gained FDA approval for the treatment of multiple myeloma (MM).

In a preclinical study, a retroviral construct of a SLAMF7-specific CAR was engineered and inserted into primary human T cells [96]. The SLAMF7–CAR T cells were tested on human MM tumor cells in vitro, ex vivo, and in orthotopic MM xenograft mouse models. The CAR T cells showed enhanced cytotoxicity against MM cell lines as well as primary MM cells. The SLAMF7 CAR T cells significantly prolonged survival of the mice xenografted with human MM.1S and IM9 myeloma cells. Another study reported construction of SLAMF7 CAR from the huLuc63 monoclonal antibody (elotuzumab) scFv [33]. The SLAMF7 CAR T cells induced rapid cytolysis of primary myeloma cells from patients with untreated and RRMM. The SLAMF7-CAR T cells were also shown to be effective in elimination of medullary and extramedullary myeloma in a murine xenograft model. These preclinical studies have paved the way for clinical translation. Two clinical phase I trials of SLAMF7-targeted CAR T cells are ongoing (NCT03710421 and 03778346).

An universal “off-the-shelf” allogeneic SLAMF7-specific CAR T cell product, UCARTCS1, was developed using TALEN-targeted gene editing [97, 98]. UCARTCS1 was tested in vitro and in mouse models against MM cell lines and primary human myeloma cells [99]. This study clearly showed that the UCARTCS1 cells could specifically target SLAMF7 and lyse MM cells both in vitro and in vivo. UCARTCS1 has been cleared by FDA for phase I study in MM patients.

A dual-target CAR construct targeting both BCMA and SLAMF7 (CS1) was also studied in vitro and in mouse models [29]. The compound CAR T cells (cCAR) contain two complete and independent CARs. the cCAR T cells were shown to have sustained in vivo activity against the MM1S cell line and induced superior survival in a mixed cell mouse model. Clinical trials are needed for these dual-target CAR T cells.

## CD138-targeted CAR T cell trial

CD138 is a surface molecule highly expressed on MM cells [100–103]. CD138 plays a significant role in the development and/or proliferation of plasma cells. CD138- targeted therapy with radioimmunoconjugate appears to be a novel approach for MM [104, 105]. A preclinical study evaluated CAR T cells targeting CD138 in vitro and in an animal model [106]. The study showed that the CAR T cells had no off-tumor toxicities. A CD138- directed CAR has been constructed with a 41BB domain [34]. The CD138- targeted CAR T cells (CART-138) were studied in five patients with RRMM in a dose-escalation phase I study (NCT01886976) (Table 2). The CD138-targeted CAR T cells were well tolerated. Four of the five patients achieved stable disease for 3 to 7 months and the CAR-T cells remained detectable by flow cytometry for more than 3 months.

**Table 2:** Non-BCMA-targeted CAR T clinical trials in multiple myeloma

NCT number (reference)	Target	Phase	Dosage	Number of patients	Responses
NCT02135406 (32)	CD19	1	1.1–6.0 × 10^8^ CAR+T cells	10	VGPR: 6; PR: 2; PD: 2
NCT01886976 (34)	CD138	1/2	0.44–1.51 × 10^7^ CAR+ T cells/kg	5	SD: 4; PD: 1
NCT00881920 (35)	κ light chain	1	0.2–2.0 × 10^8^ CAR+ T cells/m^2^	16 (7 MM)	4 SD of 7 MM

*Abbreviations: BCMA* B cell maturation antigen, *CAR* Chimeric antigen receptor, *PD* Progressive disease, *PR* Partial response, *VGPR* Very good partial response, *SD* Stable disease, *MM* Multiple myeloma

## Light chain-targeted CAR T trial

Malignant B cells are frequently light chain-restricted cells. Light chain-specific CAR (κ.CAR and lambda CAR) T cells have been engineered [35, 107]. Sixteen patients with relapsed or refractory κ + non-Hodgkin lymphoma/chronic lymphocytic leukemia (NHL/CLL) or multiple myeloma (MM) were enrolled in a phase I clinical trial of autologous κ.CAR T cells (κ.CARTs) (NCT00881920). Other treatments were discontinued in 11 of the 16 patients at least 4 weeks prior to CAR T cell infusion. The κ.CART infusion doses ranged from 0.2 to 2 × 10^8^ κ.CARTs /m^2^. κ.CART expansion was observed. Two of the 9 patients with relapsed NHL or CLL achieved CR and 1 had a PR. Four of 7 patients with RRMM remained stable for 2–17 months. The κ.CARTs were well tolerated [35].

## Conclusions and future perspectives

BCMA- targeted CAR T cells have shown high response rate in patients with RRMM with even high risk features [108]. A variety of BCMA targeted CAR T products are in active clinical development. BCMA- targeted CAR T cell product is expected to be approved for clinical therapy of RRMM soon. CAR T cells targeting CD138, CS1 glycoprotein antigen (SLAMF7) and light chains appear to be promising. CD19- targeted CAR T cells in conjunction with autologous stem cell transplantation also showed activity in RRMM. Dual- target CAR T cells are in clinical trials for RRMM. Advances in cellular immunotherapy will likely lead to significant improvement in MM therapy [30, 109–114]. In addition, most of these antigen targets are also being studied for construction of antibody-drug conjugates and bispecific antibodies [115–118]. It may be possible to combine these either concurrently or sequentially to enhance clinical efficacies.

## Data Availability

The material supporting the conclusion of this review has been included within the article.

## Abbreviations

ASCT: Autologous stem-cell transplantation
BCMA: B cell maturation antigen
CAR: Chimeric antigen receptor
CR: Complete response
IHC: Immunohistochemistry
MR: Minimal response
MRD: Minimal residual disease
nCR: Near Complete response
NR: Non-response
ORR: Overall response rate
PD: Progressive disease
PR: Partial response
RRMM: Relapsed/refractory Multiple Myeloma
sCR: Stringent complete response
SD: Stable disease
VGPR: Very good partial response

## References

[1] Avet-Loiseau H, Bahlis NJ, Chng WJ, Masszi T, Viterbo L, Pour L, Ganly P, Palumbo A, Cavo M, Langer C, Pluta A, Nagler A, Kumar S, Ben-Yehuda D, Rajkumar SV, San-Miguel J, Berg D, Lin J, van de Velde H, Esseltine DL, di Bacco A, Moreau P, Richardson PG (2017). Ixazomib significantly prolongs progression-free survival in high-risk relapsed/refractory myeloma patients. Blood.

[2] Berenson A, Vardanyan S, David M, Wang J, Harutyunyan NM, Gottlieb J, Halleluyan R, Spektor TM, Udd KA, Eshaghian S, Nassir Y, Eades B, Swift R, Berenson JR (2017). Outcomes of multiple myeloma patients receiving bortezomib, lenalidomide, and carfilzomib. Ann Hematol.

[3] Dimopoulos M, Weisel K, van de Donk N, Ramasamy K, Gamberi B, Streetly M, Offidani M, Bridoux F, de la Rubia J, Mateos MV, Ardizzoia A, Kueenburg E, Collins S, Di Micco A, Rosettani B, Li Y, Bacon P, Sonneveld P (2018). Pomalidomide plus low-dose dexamethasone in patients with relapsed/refractory multiple myeloma and renal impairment: results from a phase II trial. J Clin Oncol.

[4] Mateos MV, Dimopoulos MA, Cavo M, Suzuki K, Jakubowiak A, Knop S, Doyen C, Lucio P, Nagy Z, Kaplan P, Pour L, Cook M, Grosicki S, Crepaldi A, Liberati AM, Campbell P, Shelekhova T, Yoon SS, Iosava G, Fujisaki T, Garg M, Chiu C, Wang J, Carson R, Crist W, Deraedt W, Nguyen H, Qi M, San-Miguel J, Investigators AT (2018). Daratumumab plus Bortezomib, Melphalan, and prednisone for untreated myeloma. N Engl J Med.

[5] McCarthy PL, Owzar K, Hofmeister CC, Hurd DD, Hassoun H, Richardson PG, Giralt S, Stadtmauer EA, Weisdorf DJ, Vij R, Moreb JS, Callander NS, Van Besien K, Gentile T, Isola L, Maziarz RT, Gabriel DA, Bashey A, Landau H, Martin T, Qazilbash MH, Levitan D, McClune B, Schlossman R, Hars V, Postiglione J, Jiang C, Bennett E, Barry S, Bressler L (2012). Lenalidomide after stem-cell transplantation for multiple myeloma. N Engl J Med.

[6] Spencer A, Lentzsch S, Weisel K, Avet-Loiseau H, Mark TM, Spicka I, Masszi T, Lauri B, Levin MD, Bosi A, Hungria V, Cavo M, Lee JJ, Nooka AK, Quach H, Lee C, Barreto W, Corradini P, Min CK, Scott EC, Chanan-Khan AA, Horvath N, Capra M, Beksac M, Ovilla R, Jo JC, Shin HJ, Sonneveld P, Soong D, Casneuf T (2018). Daratumumab plus bortezomib and dexamethasone versus bortezomib and dexamethasone in relapsed or refractory multiple myeloma: updated analysis of CASTOR. Haematologica.

[7] Dimopoulos M, Wang M, Maisnar V, Minarik J, Bensinger W, Mateos M-V, Obreja M, Blaedel J, Moreau P (2018). Response and progression-free survival according to planned treatment duration in patients with relapsed multiple myeloma treated with carfilzomib, lenalidomide, and dexamethasone (KRd) versus lenalidomide and dexamethasone (Rd) in the phase III ASPIRE study. J Hematol Oncol.

[8] Majithia N, Rajkumar SV, Lacy MQ, Buadi FK, Dispenzieri A, Gertz MA, Hayman SR, Dingli D, Kapoor P, Hwa L, Lust JA, Russell SJ, Go RS, Kyle RA, Kumar SK (2016). Early relapse following initial therapy for multiple myeloma predicts poor outcomes in the era of novel agents. Leukemia.

[9] Gandolfi S, Prada CP, Richardson PG (2018). How I treat the young patient with multiple myeloma. Blood.

[10] Lonial S, Boise LH, Kaufman J (2015). How I treat high-risk myeloma. Blood.

[11] Moreau P: How I treat: New agents in myeloma. Blood 2017:blood-2017-2005-743203.

[12] June CH, O'Connor RS, Kawalekar OU, Ghassemi S, Milone MC (2018). CAR T cell immunotherapy for human cancer. Science.

[13] June CH, Sadelain M (2018). Chimeric antigen receptor therapy. N Engl J Med.

[14] Wang Z, Wu Z, Liu Y, Han W (2017). New development in CAR-T cell therapy. J Hematol Oncol.

[15] Wei G, Ding L, Wang J, Hu Y, Huang H (2017). Advances of CD19-directed chimeric antigen receptor-modified T cells in refractory/relapsed acute lymphoblastic leukemia. Exp Hematol Oncol.

[16] Zhang C, Liu J, Zhong JF, Zhang X (2017). Engineering CAR-T cells. Biomarker Res.

[17] Zhang E, Xu H (2017). A new insight in chimeric antigen receptor-engineered T cells for cancer immunotherapy. J Hematol Oncol.

[18] Liu D, Zhao J (2018). Cytokine release syndrome: grading, modeling, and new therapy. J Hematol Oncol.

[19] Porter D, Frey N, Wood PA, Weng Y, Grupp SA (2018). Grading of cytokine release syndrome associated with the CAR T cell therapy tisagenlecleucel. J Hematol Oncol.

[20] Wang Z, Han W (2018). Biomarkers of cytokine release syndrome and neurotoxicity related to CAR-T cell therapy. Biomarker Res.

[21] Neelapu SS, Tummala S, Kebriaei P, Wierda W, Gutierrez C, Locke FL, Komanduri KV, Lin Y, Jain N, Daver N, Westin J, Gulbis AM, Loghin ME, de Groot JF, Adkins S, Davis SE, Rezvani K, Hwu P, Shpall EJ (2018). Chimeric antigen receptor T-cell therapy - assessment and management of toxicities. Nat Rev Clin Oncol.

[22] Neelapu SS, Tummala S, Kebriaei P, Wierda W, Locke FL, Lin Y, Jain N, Daver N, Gulbis AM, Adkins S, Rezvani K, Hwu P, Shpall EJ (2018). Toxicity management after chimeric antigen receptor T cell therapy: one size does not fit 'ALL'. Nat Rev Clin Oncol.

[23] Grupp SA, Kalos M, Barrett D, Aplenc R, Porter DL, Rheingold SR, Teachey DT, Chew A, Hauck B, Wright JF, Milone MC, Levine BL, June CH (2013). Chimeric antigen receptor-modified T cells for acute lymphoid leukemia. N Engl J Med.

[24] Porter DL, Hwang WT, Frey NV, Lacey SF, Shaw PA, Loren AW, Bagg A, Marcucci KT, Shen A, Gonzalez V, Ambrose D, Grupp SA, Chew A, Zheng Z, Milone MC, Levine BL, Melenhorst JJ, June CH (2015). Chimeric antigen receptor T cells persist and induce sustained remissions in relapsed refractory chronic lymphocytic leukemia. Sci Transl Med.

[25] Porter DL, Kalos M, Zheng Z, Levine B, June C (2011). Chimeric antigen receptor therapy for B-cell malignancies. J Cancer.

[26] Porter DL, Levine BL, Kalos M, Bagg A, June CH (2011). Chimeric antigen receptor-modified T cells in chronic lymphoid leukemia. N Engl J Med.

[27] Turtle CJ, Hanafi LA, Berger C, Gooley TA, Cherian S, Hudecek M, Sommermeyer D, Melville K, Pender B, Budiarto TM, Robinson E, Steevens NN, Chaney C, Soma L, Chen X, Yeung C, Wood B, Li D, Cao J, Heimfeld S, Jensen MC, Riddell SR, Maloney DG (2016). CD19 CAR-T cells of defined CD4+:CD8+ composition in adult B cell ALL patients. J Clin Invest.

[28] Turtle CJ, Hanafi LA, Berger C, Hudecek M, Pender B, Robinson E, Hawkins R, Chaney C, Cherian S, Chen X, Soma L, Wood B, Li D, Heimfeld S, Riddell SR, Maloney DG (2016). Immunotherapy of non-Hodgkin's lymphoma with a defined ratio of CD8+ and CD4+ CD19-specific chimeric antigen receptor-modified T cells. Sci Transl Med.

[29] Chen KH, Wada M, Pinz KG, Liu H, Shuai X, Chen X, Yan LE, Petrov JC, Salman H, Senzel L, Leung ELH, Jiang X, Ma Y (2018). A compound chimeric antigen receptor strategy for targeting multiple myeloma. Leukemia.

[30] Ormhoj M, Bedoya F, Frigault MJ, Maus MV (2017). CARs in the Lead against multiple myeloma. Curr Hematol Malig Rep.

[31] Carpenter RO, Evbuomwan MO, Pittaluga S, Rose JJ, Raffeld M, Yang S, Gress RE, Hakim FT, Kochenderfer JN (2013). B-cell maturation antigen is a promising target for adoptive T-cell therapy of multiple myeloma. Clin Cancer Res.

[32] Garfall AL, Stadtmauer EA, Hwang WT, Lacey SF, Melenhorst JJ, Krevvata M, Carroll MP, Matsui WH, Wang Q, Dhodapkar MV, Dhodapkar K, Das R, Vogl DT, Weiss BM, Cohen AD, Mangan PA, Ayers EC, Nunez-Cruz S, Kulikovskaya I, Davis MM, Lamontagne A, Dengel K, Kerr ND, Young RM, Siegel DL, Levine BL, Milone MC, Maus MV, June CH (2018). Anti-CD19 CAR T cells with high-dose melphalan and autologous stem cell transplantation for refractory multiple myeloma. JCI Insight.

[33] Gogishvili T, Danhof S, Prommersberger S, Rydzek J, Schreder M, Brede C, Einsele H, Hudecek M (2017). SLAMF7-CAR T cells eliminate myeloma and confer selective fratricide of SLAMF7(+) normal lymphocytes. Blood.

[34] Guo B, Chen M, Han Q, Hui F, Dai H, Zhang W, Zhang Y, Wang Y, Zhu H, Han W (2016). CD138-directed adoptive immunotherapy of chimeric antigen receptor (CAR)-modified T cells for multiple myeloma. J Cell Immunother.

[35] Ramos CA, Savoldo B, Torrano V, Ballard B, Zhang H, Dakhova O, Liu E, Carrum G, Kamble RT, Gee AP, Mei Z, Wu MF, Liu H, Grilley B, Rooney CM, Brenner MK, Heslop HE, Dotti G (2016). Clinical responses with T lymphocytes targeting malignancy-associated kappa light chains. J Clin Invest.

[36] Kozlow EJ, Wilson GL, Fox CH, Kehrl JH (1993). Subtractive cDNA cloning of a novel member of the Ig gene superfamily expressed at high levels in activated B lymphocytes. Blood.

[37] Laabi Y, Gras MP, Brouet JC, Berger R, Larsen CJ, Tsapis A (1994). The BCMA gene, preferentially expressed during B lymphoid maturation, is bidirectionally transcribed. Nucleic Acids Res.

[38] Laâbi Y, Gras MP, Carbonnel F, Brouet JC, Berger R, Larsen CJ, Tsapis A (1992). A new gene, BCM, on chromosome 16 is fused to the interleukin 2 gene by a t(4;16)(q26;p13) translocation in a malignant T cell lymphoma. EMBO J.

[39] Zhou LJ, Schwarting R, Smith HM, Tedder TF (1992). A novel cell-surface molecule expressed by human interdigitating reticulum cells, Langerhans cells, and activated lymphocytes is a new member of the Ig superfamily. J Immunol.

[40] Berahovich R, Zhou H, Xu S, Wei Y, Guan J, Guan J, Harto H, Fu S, Yang K, Zhu S, Li L, Wu L, Golubovskaya V (2018). CAR-T cells based on novel BCMA monoclonal antibody block multiple myeloma cell growth. Cancers (Basel).

[41] Moreaux J, Legouffe E, Jourdan E, Quittet P, Reme T, Lugagne C, Moine P, Rossi JF, Klein B, Tarte K (2004). BAFF and APRIL protect myeloma cells from apoptosis induced by interleukin 6 deprivation and dexamethasone. Blood.

[42] Brudno JN, Maric I, Hartman SD, Rose JJ, Wang M, Lam N, Stetler-Stevenson M, Salem D, Yuan C, Pavletic S, Kanakry JA, Ali SA, Mikkilineni L, Feldman SA, Stroncek DF, Hansen BG, Lawrence J, Patel R, Hakim F, Gress RE, Kochenderfer JN (2018). T cells genetically modified to express an anti-B-cell maturation antigen chimeric antigen receptor cause remissions of poor-prognosis relapsed multiple myeloma. J Clin Oncol.

[43] Xu S, Lam KP (2001). B-cell maturation protein, which binds the tumor necrosis factor family members BAFF and APRIL, is dispensable for humoral immune responses. Mol Cell Biol.

[44] Sanchez E, Li M, Kitto A, Li J, Wang CS, Kirk DT, Yellin O, Nichols CM, Dreyer MP, Ahles CP, Robinson A, Madden E, Waterman GN, Swift RA, Bonavida B, Boccia R, Vescio RA, Crowley J, Chen H, Berenson JR (2012). Serum B-cell maturation antigen is elevated in multiple myeloma and correlates with disease status and survival. Br J Haematol.

[45] Gross JA, Johnston J, Mudri S, Enselman R, Dillon SR, Madden K, Xu W, Parrish-Novak J, Foster D, Lofton-Day C, Moore M, Littau A, Grossman A, Haugen H, Foley K, Blumberg H, Harrison K, Kindsvogel W, Clegg CH (2000). TACI and BCMA are receptors for a TNF homologue implicated in B-cell autoimmune disease. Nature.

[46] Shu HB, Johnson H (2000). B cell maturation protein is a receptor for the tumor necrosis factor family member TALL-1. Proc Natl Acad Sci U S A.

[47] Smith CA, Farrah T, Goodwin RG (1994). The TNF receptor superfamily of cellular and viral proteins: activation, costimulation, and death. Cell.

[48] Zhong Wei, Li Bo, Yang Ping, Chen Rui, Wang Cuiping, Wang Zhongqun, Shao Chen, Yuan Wei, Yan Jinchuan (2017). CD137–CD137L interaction modulates neointima formation and the phenotype transformation of vascular smooth muscle cells via NFATc1 signaling. Molecular and Cellular Biochemistry.

[49] Ali SA, Shi V, Maric I, Wang M, Stroncek DF, Rose JJ, Brudno JN, Stetler-Stevenson M, Feldman SA, Hansen BG, Fellowes VS, Hakim FT, Gress RE, Kochenderfer JN (2016). T cells expressing an anti-B-cell maturation antigen chimeric antigen receptor cause remissions of multiple myeloma. Blood.

[50] Abu-Eid R, Samara RN, Ozbun L, Abdalla MY, Berzofsky JA, Friedman KM, Mkrtichyan M, Khleif SN (2014). Selective inhibition of regulatory T cells by targeting the PI3K-Akt pathway. Cancer Immunol Res.

[51] Raje N, Berdeja J, Lin Y, Siegel D, Jagannath S, Madduri D, Liedtke M, Rosenblatt J, Maus MV, Turka A, Lam L-P, Morgan RA, Friedman K, Massaro M, Wang J, Russotti G, Yang Z, Campbell T, Hege K, Petrocca F, Quigley MT, Munshi N, Kochenderfer JN (2019). Anti-BCMA CAR T-cell therapy bb2121 in relapsed or refractory multiple myeloma. N Engl J Med.

[52] Shah N, Alsina M, Siegel DS, Jagannath S, Madduri D, Kaufman JL, Turka A, Lam LP, Massaro M, Hege K, Petrocca F, Berdeja JG, Raje N (2018). Initial results from a phase 1 clinical study of bb21217, a next-generation anti Bcma CAR T therapy. Blood.

[53] Xu J, Chen LJ, Yang SS, Sun Y, Wu W, Liu YF, Xu J, Zhuang Y, Zhang W, Weng XQ, Wu J, Wang Y, Wang J, Yan H, Xu WB, Jiang H, Du J, Ding XY, Li B, Li JM, Fu WJ, Zhu J, Zhu L, Chen Z, Fan XF, Hou J, Li JY, Mi JQ, Chen SJ (2019). Exploratory trial of a biepitopic CAR T-targeting B cell maturation antigen in relapsed/refractory multiple myeloma. Proc Natl Acad Sci U S A.

[54] Zhao W-H, Liu J, Wang B-Y, Chen Y-X, Cao X-M, Yang Y, Zhang Y-L, Wang F-X, Zhang P-Y, Lei B, Gu L-F, Wang J-L, Yang N, Zhang R, Zhang H, Shen Y, Bai J, Xu Y, Wang X-G, Zhang R-L, Wei L-L, Li Z-F, Li Z-Z, Geng Y, He Q, Zhuang Q-C, Fan FX-H, He A-L, Zhang W-G (2018). Updated analysis of a phase 1, open-label study of LCAR-B38M, a chimeric antigen receptor T cell therapy directed against B-cell maturation antigen, in patients with relapsed/refractory multiple myeloma. Blood.

[55] Zhao W-H, Liu J, Wang B-Y, Chen Y-X, Cao X-M, Yang Y, Zhang Y-L, Wang F-X, Zhang P-Y, Lei B, Gu L-F, Wang J-L, Yang N, Zhang R, Zhang H, Shen Y, Bai J, Xu Y, Wang X-G, Zhang R-L, Wei L-L, Li Z-F, Li Z-Z, Geng Y, He Q, Zhuang Q-C, Fan X-H, He A-L, Zhang W-G (2018). A phase 1, open-label study of LCAR-B38M, a chimeric antigen receptor T cell therapy directed against B cell maturation antigen, in patients with relapsed or refractory multiple myeloma. J Hematol Oncol.

[56] Mi J-Q, Fan X, Xu J, Liu Y, Zhuang Y, Yang S, Zhang W, Chen B, Wang Y, Weng X, Li J, Fu W, Jiang H, Zhu L, Chen Z, Chen S-J, Hou J (2017). Effective treatment of relapsed/refractory multiple myeloma including Extramedullary involvement by BCMA-specific chimeric antigen receptor-modified T cells. Blood.

[57] Harrington K, Wu R, Hauskins C, Amin R, Long T, Chen A, Rahardjo A, Thayer C, Navvaro G, Myers M, Jones J, Baturevych A, Morkowski S, Salmon R, Bond CJ, Staehr M, Purdon TJ, Masakayan R, Liu C, Liu H, Xu Y, Wang P, Pont M, Green DJ, Brentjens RJ, Smith EL, Sather BD (2017). Development of JCARH125: optimization of a fully human anti-Bcma CAR for use in the treatment of multiple myeloma. Blood.

[58] Mailankody S, Htut M, Lee KP, Bensinger W, Devries T, Piasecki J, Ziyad S, Blake M, Byon J, Jakubowiak A (2018). JCARH125, anti-BCMA CAR T-cell therapy for relapsed/refractory multiple myeloma: initial proof of concept results from a phase 1/2 multicenter study (EVOLVE). Blood.

[59] Jiang S, Jin J, Hao S, Yang M, Chen L, Ruan H, Xiao J, Wang W, Li Z, Yu K (2018). Low dose of human scFv-derived BCMA-targeted CAR-T cells achieved fast response and high complete remission in patients with relapsed/refractory multiple myeloma. Blood.

[60] Smith EL, Staehr M, Masakayan R, Tatake IJ, Purdon TJ, Wang X, Wang P, Liu H, Xu Y, Garrett-Thomson SC, Almo SC, Riviere I, Liu C, Brentjens RJ (2018). Development and evaluation of an optimal human single-chain variable fragment-derived BCMA-targeted CAR T cell vector. Mol Ther.

[61] Mailankody S, Ghosh A, Staehr M, Purdon TJ, Roshal M, Halton E, Diamonte C, Pineda J, Anant P, Bernal Y, Wills A, Korde N, Lendvai N, Lesokhin AM, Hassoun H, Hultcrantz M, Landau HJ, Shah GL, Scordo M, Chung DJ, Lahoud OB, Khattar P, Fernandez de Larrea C, Gao Q, Jungbluth A, Park JH, Curran KJ, Sauter CS, Palomba ML, Senechal B (2018). Clinical Responses and Pharmacokinetics of MCARH171, a Human-Derived Bcma Targeted CAR T Cell Therapy in Relapsed/Refractory Multiple Myeloma: Final Results of a Phase I Clinical Trial. Blood.

[62] Li C, Wang Q, Zhu H, Mao X, Wang Y, Zhang Y, Zhou J (2018). T cells expressing anti B-cell maturation antigen chimeric antigen receptors for plasma cell malignancies. Blood.

[63] Xu J, Wang Q, Xu H, Gu C, Jiang L, Wang J, Wang D, Xu B, Mao X, Wang J, Wang Z, Xiao Y, Zhang Y, Li C, Zhou J (2018). Anti-BCMA CAR-T cells for treatment of plasma cell dyscrasia: case report on POEMS syndrome and multiple myeloma. J Hematol Oncol.

[64] Li C, Zhou J, Wang J, Hu G, Du A, Zhou X, Meng L, Hong Z, Chen L, Mao X (2019). Clinical responses and pharmacokinetics of fully human BCMA targeting CAR T-cell therapy in relapsed/refractory multiple myeloma. J Clin Oncol.

[65] Cohen AD, Garfall AL, Stadtmauer EA, Melenhorst JJ, Lacey SF, Lancaster E, Vogl DT, Weiss BM, Dengel K, Nelson A, Plesa G, Chen F, Davis MM, Hwang WT, Young RM, Brogdon JL, Isaacs R, Pruteanu-Malinici I, Siegel DL, Levine BL, June CH, Milone MC (2019). B cell maturation antigen-specific CAR T cells are clinically active in multiple myeloma. J Clin Invest.

[66] Zhao S, Jiang E, Chen S, Gu Y, Shangguan AJ, Lv T, Luo L, Yu Z (2016). PiggyBac transposon vectors: the tools of the human gene encoding. Transl Lung Cancer Res.

[67] Gregory T, Cohen AD, Costello CL, Ali SA, Berdeja JG, Ostertag EM, Martin C, Shedlock DJ, Resler ML, Spear MA, Orlowski RZ, Patel KK (2018). Efficacy and safety of P-Bcma-101 CAR-T cells in patients with relapsed/refractory (r/r) multiple myeloma (MM). Blood.

[68] Lee DH, Cervantes-Contreras F, Lee SY, Green DJ, Till BG (2018). Improved expansion and function of CAR T cell products from cultures initiated at defined CD4:CD8 ratios. Blood.

[69] Green DJ, Pont M, Sather BD, Cowan AJ, Turtle CJ, Till BG, Nagengast AM, Libby EN, Becker PS, Coffey DG, Tuazon SA, Wood B, Blake M, Works M, Thompson BS, Gooley T, Appelbaum FR, Maloney DG, Riddell SR (2018). Fully human Bcma targeted chimeric antigen receptor T cells administered in a defined composition demonstrate potency at low doses in advanced stage high risk multiple myeloma. Blood.

[70] Locke FL, Neelapu SS, Bartlett NL, Siddiqi T, Chavez JC, Hosing CM, Ghobadi A, Budde LE, Bot A, Rossi JM, Jiang Y, Xue AX, Elias M, Aycock J, Wiezorek J, Go WY (2017). Phase 1 results of ZUMA-1: a multicenter study of KTE-C19 anti-CD19 CAR T cell therapy in refractory aggressive lymphoma. Mol Ther.

[71] Maude SL, Frey N, Shaw PA, Aplenc R, Barrett DM, Bunin NJ, Chew A, Gonzalez VE, Zheng Z, Lacey SF, Mahnke YD, Melenhorst JJ, Rheingold SR, Shen A, Teachey DT, Levine BL, June CH, Porter DL, Grupp SA (2014). Chimeric antigen receptor T cells for sustained remissions in leukemia. N Engl J Med.

[72] Schuster SJ, Svoboda J, Nasta SD, Chong EA, Winchell N, Landsburg DJ, Porter DL, Mato AR, Strauser HT, Schrank-Hacker AM, Wasik MA, Lacey SF, Melenhorst JJ, Chew A, Hasskarl J, Marcucci KT, Levine BL, June CH (2016). Treatment with Chimeric Antigen Receptor Modified T Cells Directed Against CD19 (CTL019) Results in Durable Remissions in Patients with Relapsed or Refractory Diffuse Large B Cell Lymphomas of Germinal Center and Non-Germinal Center Origin, "Double Hit" Diffuse Large B Cell Lymphomas, and Transformed Follicular to Diffuse Large B Cell Lymphomas. Blood.

[73] Schuster SJ (2019). CD19-directed CAR T cells gain traction. Lancet Oncol.

[74] Schuster SJ, Bishop MR, Tam CS, Waller EK, Borchmann P, McGuirk JP, Jäger U, Jaglowski S, Andreadis C, Westin JR, Fleury I, Bachanova V, Foley SR, Ho PJ, Mielke S, Magenau JM, Holte H, Pantano S, Pacaud LB, Awasthi R, Chu J, Anak Ö, Salles G, Maziarz RT (2018). Tisagenlecleucel in adult relapsed or refractory diffuse large B-cell lymphoma. N Engl J Med.

[75] Sadelain M, Brentjens R, Riviere I, Park J (2015). CD19 CAR therapy for acute lymphoblastic leukemia. Am Soc Clin Oncol Educ Book.

[76] Neelapu SS, Locke FL, Bartlett NL, Lekakis L, Miklos D, Jacobson CA, Braunschweig I, Oluwole O, Siddiqi T, Lin Y, Timmerman J, Stiff PJ, Friedberg J, Flinn I, Goy A, Smith M, Deol A, Farooq U, McSweeney P, Munoz J, Avivi I, Castro JE, Westin JR, Chavez JC, Ghobadi A, Komanduri KV, Levy R, Jacobsen ED, Reagan P, Bot A (2016). Kte-C19 (anti-CD19 CAR T Cells) Induces Complete Remissions in Patients with Refractory Diffuse Large B-Cell Lymphoma (DLBCL): Results from the Pivotal Phase 2 Zuma-1. Blood.

[77] Neelapu SS, Locke FL, Bartlett NL, Lekakis LJ, Miklos DB, Jacobson CA, Braunschweig I, Oluwole OO, Siddiqi T, Lin Y, Timmerman JM, Stiff PJ, Friedberg JW, Flinn IW, Goy A, Hill BT, Smith MR, Deol A, Farooq U, McSweeney P, Munoz J, Avivi I, Castro JE, Westin JR, Chavez JC, Ghobadi A, Komanduri KV, Levy R, Jacobsen ED, Witzig TE (2017). Axicabtagene Ciloleucel CAR T-cell therapy in refractory large B-cell lymphoma. N Engl J Med.

[78] Del Nagro CJ, Otero DC, Anzelon AN, Omori SA, Kolla RV, Rickert RC (2005). CD19 function in central and peripheral B-cell development. Immunol Res.

[79] Garfall AL, Maus MV, Hwang WT, Lacey SF, Mahnke YD, Melenhorst JJ, Zheng Z, Vogl DT, Cohen AD, Weiss BM, Dengel K, Kerr ND, Bagg A, Levine BL, June CH, Stadtmauer EA (2015). Chimeric antigen receptor T cells against CD19 for multiple myeloma. N Engl J Med.

[80] Boyer LA, Lee TI, Cole MF, Johnstone SE, Levine SS, Zucker JP, Guenther MG, Kumar RM, Murray HL, Jenner RG, Gifford DK, Melton DA, Jaenisch R, Young RA (2005). Core transcriptional regulatory circuitry in human embryonic stem cells. Cell.

[81] Tai YT, Chang BY, Kong SY, Fulciniti M, Yang G, Calle Y, Hu Y, Lin J, Zhao JJ, Cagnetta A, Cea M, Sellitto MA, Zhong MY, Wang Q, Acharya C, Carrasco DR, Buggy JJ, Elias L, Treon SP, Matsui W, Richardson P, Munshi NC, Anderson KC (2012). Bruton tyrosine kinase inhibition is a novel therapeutic strategy targeting tumor in the bone marrow microenvironment in multiple myeloma. Blood.

[82] Tanno T, Lim Y, Wang Q, Chesi M, Bergsagel PL, Matthews G, Johnstone RW, Ghosh N, Borrello I, Huff CA, Matsui W (2014). Growth differentiating factor 15 enhances the tumor-initiating and self-renewal potential of multiple myeloma cells. Blood.

[83] Feng K-C, Guo Y-I, Liu Y, Dai H-R, Wang Y, Lv H-Y, Huang J-H, Yang Q-M, Han W-D (2017). Cocktail treatment with EGFR-specific and CD133-specific chimeric antigen receptor-modified T cells in a patient with advanced cholangiocarcinoma. J Hematol Oncol.

[84] Huang L, Wang N, Cao Y, Li C, Xiao Y, Xiao M, Zhou X, Wang G, Hong Z, Zhen M, Meng W, Zhang B, Zhang Y, Marcucci G, Zhang T, Zhou J (2018). CAR22/19 cocktail therapy for patients with refractory/relapsed B-cell malignancies. Blood.

[85] Liu S, Deng B, Lin Y, Yin Z, Pan J, Wu T, Gao Z, Song Y, Zhao Y, Tong C (2018). Sequential CD19- and CD22-CART cell therapies for relapsed B-cell acute lymphoblastic leukemia after allogeneic hematopoietic stem cell transplantation. Blood.

[86] Yan L, Shang J, Kang L, Shi X, Zhou J, Jin S, Yao W, Yao Y, Chen G, Zhu Z, Chang H, Wu D, Yu L, Fu C (2017). Combined infusion of CD19 and Bcma-specific chimeric antigen receptor T cells for RRMM: initial safety and efficacy report from a clinical pilot study. Blood.

[87] Shi X, Yan L, Shang J, Qu S, Kang L, Zhou J, Jin S, Yao W, Yao Y, Yan S, Liu Y, Chen G, Zhu Z, Chang H, Wu D, Yu L, Fu C (2018). Tandom autologous transplantation and combined infusion of CD19 and Bcma-specific chimeric antigen receptor T cells for high risk MM: initial safety and efficacy report from a clinical pilot study. Blood.

[88] Hsi ED, Steinle R, Balasa B, Szmania S, Draksharapu A, Shum BP, Huseni M, Powers D, Nanisetti A, Zhang Y, Rice AG, van Abbema A, Wong M, Liu G, Zhan F, Dillon M, Chen S, Rhodes S, Fuh F, Tsurushita N, Kumar S, Vexler V, Shaughnessy JD, Barlogie B, van Rhee F, Hussein M, DEH A, Williams MB (2008). CS1, a potential new therapeutic antibody target for the treatment of multiple myeloma. Clin Cancer Res.

[89] Elmaagacli AH, Salwender H, Jehn C, Dahmash F, Singh A, Wilson O, Pannenbeckers M, Niggemann C, Vierbuchen M. Strong expression of SLAMF7 in natural killer/T-cell lymphoma and large granular lymphocyte leukemia - a prominent biomarker and potential target for anti-SLAMF7 antibody therapy. Leuk Lymphoma. 2019;60:1–4. 10.1080/10428194.2019.1623887 31164030

[90] Bouwstra R, van Meerten T, Bremer E (2019). Does cancer cell-expressed SLAMF7 impact on CD47-mediated phagocytosis?. Mol Cell Oncol.

[91] Ashour R, Ri M, Aly SS, Yoshida T, Tachita T, Kanamori T, Aoki S, Kinoshita S, Narita T, Totani H, Masaki A, Ito A, Kusumoto S, Komatsu H, Mansour S, Elsaied AA, Iida S (2019). Expression analysis of two SLAM family receptors, SLAMF2 and SLAMF7, in patients with multiple myeloma. Int J Hematol.

[92] He Y, Bouwstra R, Wiersma VR, de Jong M, Jan Lourens H, Fehrmann R, de Bruyn M, Ammatuna E, Huls G, van Meerten T, Bremer E (2019). Cancer cell-expressed SLAMF7 is not required for CD47-mediated phagocytosis. Nat Commun.

[93] O'Connell P, Pepelyayeva Y, Blake MK, Hyslop S, Crawford RB, Rizzo MD, Pereira-Hicks C, Godbehere S, Dale L, Gulick P, Kaminski NE, Amalfitano A, Aldhamen YA (2019). SLAMF7 is a critical negative regulator of IFN-alpha-mediated CXCL10 production in chronic HIV infection. J Immunol.

[94] Shi J, Bodo J, Zhao X, Durkin L, Goyal T, Meyerson H, Hsi ED (2019). SLAMF7 (CD319/CS1) is expressed in plasmablastic lymphoma and is a potential diagnostic marker and therapeutic target. Br J Haematol.

[95] Dimopoulos MA, Dytfeld D, Grosicki S, Moreau P, Takezako N, Hori M, Leleu X, LeBlanc R, Suzuki K, Raab MS, Richardson PG, Popa McKiver M, Jou Y-M, Shelat SG, Robbins M, Rafferty B, San-Miguel J (2018). Elotuzumab plus Pomalidomide and dexamethasone for multiple myeloma. N Engl J Med.

[96] Chu J, He S, Deng Y, Zhang J, Peng Y, Hughes T, Yi L, Kwon C-H, Wang Q-E, Devine SM, He X, Bai X-F, Hofmeister CC, Yu J (2014). Genetic modification of T cells redirected toward CS1 enhances eradication of myeloma cells. Clin Cancer Res.

[97] Zhao J, Lin Q, Song Y, Liu D (2018). Universal CARs, universal T cells, and universal CAR T cells. J Hematol Oncol.

[98] Zhao J, Song Y, Liu D (2019). Clinical trials of dual-target CAR T cells, donor-derived CAR T cells, and universal CAR T cells for acute lymphoid leukemia. J Hematol Oncol.

[99] Mathur R, Zhang Z, He J, Galetto R, Gouble A, Chion-Sotinel I, Filipe S, Gariboldi A, Veeramachaneni T, Manasanch EE, Thomas SK, Lee HC, Patel KK, Weber DM, Davis RE, Orlowski RJ, Smith J, Yang J, Neelapu SS (2017). Universal SLAMF7-specific CAR T-cells as treatment for multiple myeloma. Blood.

[100] Storti Paola, Agnelli Luca, Palma Benedetta dalla, Todoerti Katia, Marchica Valentina, Accardi Fabrizio, Sammarelli Gabriella, Deluca Federica, Toscani Denise, Costa Federica, Vicario Emanuela, Todaro Giannalisa, Martella Eugenia, Neri Antonino, Giuliani Nicola (2019). The transcriptomic profile of CD138+ cells from patients with early progression from smoldering to active multiple myeloma remains substantially unchanged. Haematologica.

[101] Liu Zhao‐Yun, Tian Meng‐Yue, Deng Ling, Wang Ying‐Shuai, Xing Rui, Liu Hui, Fu Rong (2019). The potential diagnostic power of CD138+ microparticles from the plasma analysis for multiple myeloma clinical monitoring. Hematological Oncology.

[102] Mykytiv V, Alwaheed A, Mohd Hashim NA (2019). Double CD38(−)/CD138(−) negative multiple myeloma. Hematol Oncol Stem Cell Ther.

[103] Dass J, Arava S, Mishra PC, Dinda AK, Pati HP (2019). Role of CD138, CD56, and light chain immunohistochemistry in suspected and diagnosed plasma cell myeloma: a prospective study. South Asian J Cancer.

[104] Fichou N, Gouard S, Maurel C, Barbet J, Ferrer L, Morgenstern A, Bruchertseifer F, Faivre-Chauvet A, Bigot-Corbel E, Davodeau F, Gaschet J, Chérel M (2015). Single-dose anti-CD138 Radioimmunotherapy: Bismuth-213 is more efficient than Lutetium-177 for treatment of multiple myeloma in a preclinical model. Frontiers Med.

[105] Gouard S, Pallardy A, Gaschet J, Faivre-Chauvet A, Bruchertseifer F, Morgenstern A, Maurel C, Matous E, Kraeber-Bodere F, Davodeau F, Cherel M (2014). Comparative analysis of multiple myeloma treatment by CD138 antigen targeting with bismuth-213 and Melphalan chemotherapy. Nucl Med Biol.

[106] Sun C, Mahendravada A, Ballard B, Kale B, Ramos C, West J, Maguire T, McKay K, Lichtman E, Tuchman S, Dotti G, Savoldo B (2019). Safety and efficacy of targeting CD138 with a chimeric antigen receptor for the treatment of multiple myeloma. Oncotarget.

[107] Ranganathan R (2018). Chimeric antigen receptor T cells targeting the lambda light chain of human immunoglobulin as a viable target for B cell non-Hodgkin lymphoma. J Clin Oncol.

[108] Greipp PR, San Miguel J, Durie BG, Crowley JJ, Barlogie B, Blade J (2005). International staging system for multiple myeloma. J Clin Oncol.

[109] Sadelain M, Rivière I, Riddell S (2017). Therapeutic T cell engineering. Nature.

[110] Wang J, Hu Y, Huang H (2018). Current development of chimeric antigen receptor T-cell therapy. Stem Cell Investigation.

[111] Patel S, Burga RA, Powell AB, Chorvinsky EA, Hoq N, McCormack SE, Van Pelt SN, Hanley PJ, Cruz CRY. Beyond CAR T Cells: Other Cell-Based Immunotherapeutic Strategies Against Cancer. Frontiers Oncol. 2019;9(196). 10.3389/fonc.2019.00196. 10.3389/fonc.2019.00196 PMC6467966 31024832

[112] Cohen AD: CAR T Cells and Other Cellular Therapies for Multiple Myeloma: 2018 Update. Am Soc Clin Oncol Educ Book. 2018;38:e6-e15. 10.1200/EDBK_200889 30231373

[113] Pang Y, Hou X, Yang C, Liu Y, Jiang G (2018). Advances on chimeric antigen receptor-modified T-cell therapy for oncotherapy. Mol Cancer.

[114] Ye B, Stary CM, Li X, Gao Q, Kang C, Xiong X (2018). Engineering chimeric antigen receptor-T cells for cancer treatment. Mol Cancer.

[115] Aujla A, Aujla R, Liu D (2019). Inotuzumab ozogamicin in clinical development for acute lymphoblastic leukemia and non-Hodgkin lymphoma. Biomarker Res.

[116] Peters C, Brown S (2015). Antibody-drug conjugates as novel anti-cancer chemotherapeutics. Biosci Rep.

[117] Kantarjian HM, DeAngelo DJ, Stelljes M, Martinelli G, Liedtke M, Stock W, Gokbuget N, O'Brien S, Wang K, Wang T, Paccagnella ML, Sleight B, Vandendries E, Advani AS (2016). Inotuzumab Ozogamicin versus standard therapy for acute lymphoblastic leukemia. N Engl J Med.

[118] Kantarjian H, Stein A, Gökbuget N, Fielding AK, Schuh AC, Ribera J-M, Wei A, Dombret H, Foà R, Bassan R, Arslan Ö, Sanz MA, Bergeron J, Demirkan F, Lech-Maranda E, Rambaldi A, Thomas X, Horst H-A, Brüggemann M, Klapper W, Wood BL, Fleishman A, Nagorsen D, Holland C, Zimmerman Z, Topp MS (2017). Blinatumomab versus chemotherapy for advanced acute lymphoblastic leukemia. N Engl J Med.

